# Piecing Together a Puzzle of Exceptional Lesions: A Retrospective Study of a Potpourri of 160 Space-Occupying Lesions of the Central Nervous System

**DOI:** 10.7759/cureus.23585

**Published:** 2022-03-28

**Authors:** Charusheela R Gore, Pratyush Mishra, Rakesh Rashmi, Ashish Chugh

**Affiliations:** 1 Pathology, Dr. D. Y. Patil Medical College, Hospital & Research Centre, Dr. D. Y. Patil Vidyapeeth, Pune, IND; 2 Neurosurgery, Dr. D. Y. Patil Medical College, Hospital & Research Centre, Dr. D. Y. Patil Vidyapeeth, Pune, IND

**Keywords:** space-occupying lesions, histopathology examination, brain tumors cns tumors, meningioma, astrocytoma, tumor, cns

## Abstract

Introduction

Central nervous system (CNS) lesions are rare and histologically heterogenous, and carry serious potential for patient morbidity and mortality. A retrospective epidemiological review of CNS neoplasms is of great importance for future research because it can demonstrate the changes in the spectrum of CNS lesions of a population, unveil the possible associated risk factors, and indicate the potential therapeutic methods for various neoplastic and non-neoplastic lesions. Neurosurgeons have always shown an obsession with a good neuropathological diagnosis in intracranial and extracranial lesions. This obsession need not be overemphasized as it helps the clinician plan an adequate surgical/treatment strategy to optimize outcomes and minimize morbidity.

Methods

This study included a spectrum of 160 biopsies of patients with space-occupying lesions of the CNS during a period of two years (2019-2021). All the cases were studied and analyzed, and their histological typing/grading was done. The cases were graded and categorized according to the 2016 WHO Classification of CNS Tumors.

Results

Among 160 cases, the study showed a slight male preponderance of 100 (62.5%) cases. The maximum number of cases, 37 (23%) cases, was in the age group of 41-50 years. Clinically, the commonest complaints were headache and seizures. The most common location of tumor was supra-tentorial, comprising around 96 (60%) cases, of which 27 (28%) cases were located in the frontal lobe. There were four (2.5%) cases that had non-neoplastic lesions and the rest 156 (97.5%) cases had neoplastic lesions. Malignant lesions outnumbered the benign lesions, comprising of 82 (51.25%) cases. Among the neoplastic lesions, the highest cases were of astrocytoma, 48 (30.76%) cases, followed by meningioma, 42 (26.92%) cases. Also, 21 extremely rare and unusual cases were encountered.

Conclusion

The present study reflects the diversity of histopathological spectrum of CNS lesions in our center. In-depth studies from across various hospitals are required to have representative data on the incidence, epidemiological profile, and etiology of CNS lesions in India.

## Introduction

Central nervous system space-occupying lesions (CNS SOLs) are defined as any lesion in the cranium with varied etiology such as infectious, neoplastic, vascular malformation, or inflammatory. CNS SOLs are exceptionally heterogenous histologically and are mightily fatal, thus accounting for substantial morbidity and mortality worldwide. Malignant and non-malignant CNS tumors contain a diverse constellation of more than 100 histological subtypes with distinct epidemiology, clinical features, treatments, and outcomes [1,2]. The annual incidence of CNS tumors ranges from 10 to 17 per 100,000 for intracranial tumors and -one to two per 100,000 for intraspinal tumors, whereas in India they constitute around 1.9% of all tumors [2,3]. Formerly, the incidence of brain tumors in India was found to be uncommon when compared to western countries, but in the last 20 years, studies have revealed that these lesions are as common in India as elsewhere. Because of a lag in resources and expertise in neuropathology, various challenges pop up in diagnosing cases with rare histology, uncommon location, and unfitting age group. Thus, the uniqueness of the lesions and tumor burden remain understated.

Therefore, tertiary hospital based studies for various parameters, most importantly various histological subtyping, solve this riddle and create a pathway for timely and prompt neurosurgical intervention [2,4].

This article provides an overview of non-malignant lesions, neoplastic lesions, benign and malignant CNS tumor incidence, different histological subtypes, anatomic site, sex, and varying clinical features, along with 21 unexampled lesions that are sparsely encountered and documented.

## Materials and methods

This study included a spectrum of 160 biopsies of patients with SOLs of CNS admitted to the Department of Neurosurgery during a period of two years (2019-2021). The sample size included all 160 biopsies (N=160). All surgical sample of suspected CNS neoplasm operated in the Department of Neurosurgery were received in the Department of Pathology. The tissue was fixed with 10% formalin and processed in graded alcohol and xylene, and then paraffin blocking and cutting was performed according to standard guideline and stained with routine H&E stain. The cases were diagnosed, graded, and subtyped with immunohistochemistry (IHC) wherever needed as well. The inclusion criteria contained cases of CNS SOLs of all age groups from the brain and spinal cord. All the cases were studied and analyzed, and their histological typing/grading was done. The cases were graded and categorized according to the 2016 WHO Classification of CNS Tumors.

## Results

The department received a total of 160 cases of SOLs of the brain and spinal cord. It showed a slight male preponderance 100 (62.5%) cases in the group (Table 1).

**Table 1:** Characteristics of 160 biopsies in spectrum (N=160)

Characteristics	N (%)
Age (years)	
0 -10	7 (4.37%)
11-20	13 (8.125%)
21-30	22 (13.75%)
31-40	28 (17.5%)
41-50	37 (23.125%)
51-60	31 (19.35%)
61-70	19 (11.87%)
71 and above	3 (1.87%)
Gender	
Male	100 (62.5%)
Female	60 (37.5%)
Clinical manifestations	
Headaches	53 (33%)
Seizures	46 (29%)
Vomiting	21 (13%)
Neurological deficit	16 (10%)
Altered sensorium	16 (10%)
Blurring of vision	5 (3%)
Other	3 (2%)
Location	
Supratentorial	96 (60%)
Infratentorial	64 (40%)
Topography of lesions	
Frontal	45 (28%)
Temporoparietal	24 (15%)
Frontotemporal	18 (11%)
Choroid plexus	16 (10%)
Sellarsupra sellar	13 (8%)
Spinal cord	14 (9%)
Parietal	9 (6%)
Brain stem	13 (8%)
Other	8 (5%)

The maximum number of cases, 37 (23%) cases, was seen in the age group of 41-50 years, followed by 51-60 years and 31-40 years age group, comprising of 31 (19%) cases and 28 (17.5%) cases, respectively (Table 1).

Clinically, the commonest complaints in our patients were headache and seizures, constituting around 62% cases, followed by vomiting, altered sensorium, and neurological deficit (Table 1). Visual disturbances were seen in around 3% of the cases. Also, some of the pediatric patients presented with hydrocephalus.

The most common location of tumor was supratentorial, comprising around 96 (60%) cases, among which 27 (28%) cases were located in the frontal lobe and 14 (15%) in the temporoparietal lobe (Table 1). Around 64 (40%) cases were located in the infratentorial region, mainly in the brain stem and spinal cord region.

**Figure 1:**
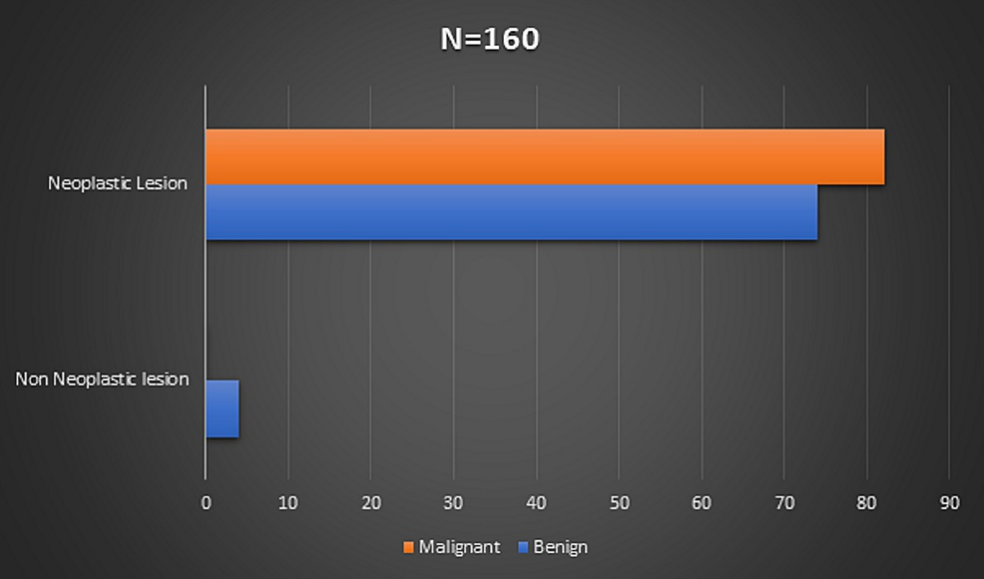
Non-neoplastic:neoplastic lesion ratio (N=160)

Among 160 cases, there were four (2.5%) cases of non-neoplastic lesions, comprising of epidermoid cyst, granulomatous lesion, and lipomeningomyelocele. The rest 156 cases were of neoplastic lesions. Malignant lesions outnumbered the benign lesions, comprising of 82 (51.25%) cases. The malignant lesions constituted 74 (46.25%) cases (Figure 1).

**Figure 2:**
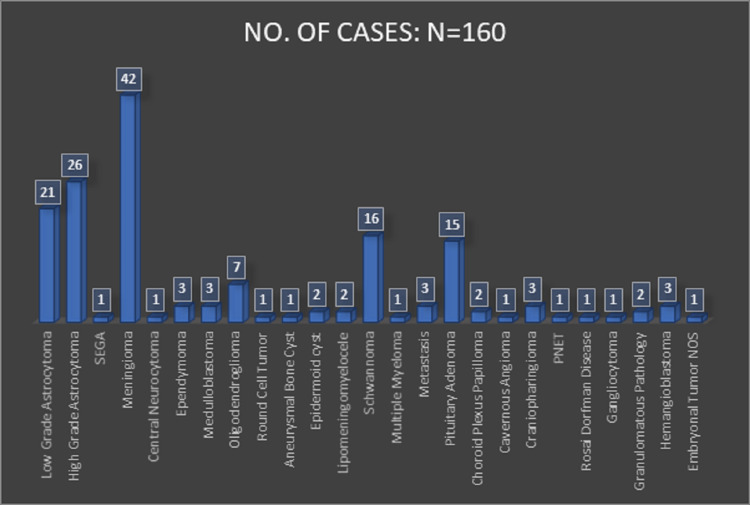
: Incidence of lesions (N=160)

Among the neoplastic lesions, the highest number of cases was of astrocytoma, 48 (30.76%) cases, followed by meningioma, 42 (26.92%) cases. Also included were cases of metastatic deposits, schwannoma, choroid plexus papilloma, medulloblastoma, adenoid cystic carcinoma of orbit, ependymoma, embryonal tumor nos metastasis, pituitary adenoma, and craniopharyngioma (Figure 2).

The relative frequency of each lesion was also calculated (Table 2).

**Table 2:** Relative frequency of tumor categories (N=160) PNET, primitive neuroectodermal tumor; NOS, not otherwise specified

Histological type	Total cases, N (%)
Astrocytoma (low-grade astrocytoma and high-grade astrocytoma)	48 (30%)
Meningioma	42 (26.25%)
Schwannoma	16 (10%)
Oligodendroglioma	7 (4.37%)
Ependymoma	3 (1.87%)
Medulloblastoma	3 (1.87%)
Pituitary adenoma	15 (9.37%)
Craniopharyngioma	3 (1.87%)
Choroid plexus papilloma	2 (1.25%)
Epidermoid cyst	2 (1.25%)
Metastasis	3 (1.87%)
Round cell tumor (adenoid cystic carcinoma of orbit)	1 (0.62%)
Aneurysmal bone cyst	1 (0.62%)
Lipomeningomyelocele	2 (1.25%)
Hemangioblastoma	3 (1.87%)
Sinonasal carcinoma encroaching cerebral cortex	1 (0.62%)
Multiple myeloma	1 (0.62%)
Cavernous angioma	1 (0.62%)
PNET	1 (0.62%)
Extradural Rosai-Dorfman disease	1 (0.62%)
Gangliocytoma	1 (0.62%)
Granulomatous pathology	2 (1.25%)
Central neurocytoma	1 (0.62%)
Embryonal tumor NOS	1 (0.62%)
Total	160 (100%)

Extremely rare and unusual cases of extradural Rosai-Dorfman disease, primitive neuroectodermal tumor (PNET) of D4-D5, subependymal giant cell astrocytoma (SEGA), adenoid cystic carcinoma of orbit, ganglioglioma, anaplastic oligodendroglioma, gangliocytoma with pituitary adenoma, psammomatous meningioma at the level of thoracic vertebrae, meningioma with rhabdoid differentiation, gliosarcoma, anaplastic meningioma, metastatic squamous cell carcinoma, IDH-mutated anaplastic astrocytoma with clear cell morphology, cavernous angioma, embryonal tumor not otherwise specified (NOS), mucin-secreting metastatic carcinoma, embryonal CNS tumor NOS, and few other rare entities were also diagnosed (Table 3).

**Table 3:** Representation of unusual cases *Indicates a provisional clinical diagnosis CNS, central nervous system; SOL, space-occupying lesion; SEGA, subependymal giant cell astrocytoma; PNET, primitive neuroectodermal tumor; NOS, not otherwise specified

Sl. no	Age/sex	Clinical diagnosis	Final diagnosis
1	60/M	High-grade glioma*	Gliosarcoma
2	26/M	High-grade glioma*	CNS sarcoidosis
3	63/M	High-grade glioma*	Metastasis of mucin-secreting carcinoma
4	18/M	Meningioma*	Cavernous angioma
5	31/M	Neoplastic etiology*	IDH-mutated anaplastic astrocytoma with clear cell morphology
6	40/M	Ependymoma*, Medulloblastoma*	Central neurocytoma
7	41/F	Extramedullary SOL	Multiple myeloma
8	36/M	Neoplastic etiology*	Adenoid cystic carcinoma of the orbit
9	74/M	Meningioma*	Anaplastic meningioma
10	23/M	Low-grade glioma*	Ganglioglioma/mixed glial tumor
11	51/F	Recurrence tumor	Anaplastic oligodendroglioma
12	43/M	Pituitary adenoma	Gangliocytoma with pituitary adenoma
13	40/F	Meningioma*	Thoracic psammomatous spinal meningioma at T3-T4
14	36/M	Meningioma*	Meningioma with rhabdoid differentiation
15	10/F	High-grade glioma*	Ventricular SEGA
16	36/M	High-grade glioma*	Metastatic squamous cell carcinoma
17	55/M	High-grade glioma*	Gliosarcoma
18	68/M	Extradural multiple myeloma*	Extranodal intradural Rosai-Dorfman disease
19	45/F	Schwannoma*	PNET D4-D5
20	14/M	Pilocytic astrocytoma*	Post-pubescent medulloblastoma
21	2/M	High-grade glioma*	CNS embryonal tumor NOS

A vertebral lesion put us in a diagnostic dilemma for multiple pathologies, where the 68-year-old male presented to us without lymphadenopathy, with D7-D8 extradural lytic lesion and epidural mass causing cord compression, with raised serum protein levels on serum electrophoresis. The patient was confused as a case of multiple myeloma, which was later on diagnosed as extranodal intradural Rosai-Dorfman disease.

A 45-year-old female patient presented with pain in the thoracic region, bilateral lower limb weakness, and bladder and bowel dysfunction. MRI of the thoracic spine revealed an L2-L4 extradural SOL. An L2-L4 Laminectomy and transversectomy were performed. Histopathology examination confirmed the diagnosis of PNET.

A 40-year-old female who presented with progressive bilateral lower limb weakness, urinary incontinence for two months, and inability to walk for 15 days was identified histologically as a case of psammomatous meningioma.

We also received a biopsy from a 36-year-old male who presented with chronic headache and two episodes of seizures who was further labelled as a rare case of atypical meningioma with rhabdoid differentiation.

A 23-year-old male who was misdiagnosed as a case of anaplastic astrocytoma on intraoperative cytodiagnosis during frozen section was diagnosed as a case of ganglioglioma on final histopathology report and IHC study.

Another 51-year-old female who presented with unilateral facial palsy, a previously operated case of oligodendroglioma who also received four cycles of chemotherapy and radiotherapy four years prior to presentation, was identified as a case of anaplastic oligodendroglioma on histopathology.

An 18-year-old male presented to the Neurosurgery Outpatient Department with a history of seizures for three years. The patient underwent plain axial computed tomography (CT) scan, which revealed cortex-based lesion in the parafalcine right anterior frontal region suspecting a neoplastic etiology. A histopathology diagnosis of cavernous angioma mixed or hybrid type was made.

An eight-year-old female with a known medical history of tuberous sclerosis presented with recurrent headaches. Radiological workup revealed a homogenously enhancing mass in the left lateral ventricle with eccentric calcification and obstructive hydrocephalus. Final histopathology report concluded with a final diagnosis of SEGA.

A 36-year-old female patient presented with headache and unilateral weakness for a month. MRI of the brain revealed a dural-based extra axial solid T2 hypo-intense lesion in the right frontoparietal region, and a diagnosis of meningioma was made. Yet, histopathology and raised angiotensin-converting enzyme (ACE) levels confirmed a diagnosis of sarcoidosis.

A 48-year-old male presented with chronic headache and episodes of generalized tonic-clonic seizures for four months. Radiologic investigation revealed a complex left parieto-occipital heterogeneous mass lesion with cystic and solid components. Histopathology and IHC confirmed gliosarcoma.

A two-year-old female presented with generalized tonic-clonic seizures for three days. Radiological examination revealed high-grade cerebellar lesion. Final diagnosis with histopathology and IHC confirmed a CNS embryonal tumor NOS.

## Discussion

A retrospective epidemiological review of CNS SOLs is of utmost importance as it can demonstrate the changes in the spectrum of these lesions in a population, reveal the varied topography and possible associated risk factors, and also suggest a potential therapy method for these lesions.

In the current study, attempts were made to document the spectrum of CNS tumor according to the 2016 WHO Classification of CNS Tumors. Out of the 160 CNS lesions, neoplastic outnumbered the non-neoplastic lesions. Other studies in India conducted by Khonglah et al. and Naik et al. showed 86.13% versus 13.87% and 91% versus 9% distribution of lesions of neoplastic versus non-neoplastic pathology, respectively [2,5,6].

Only four lesions were non-neoplastic, comprising of epidermoid cyst, granulomatous lesion, and lipomeningomyelocele. The rest 156 cases were neoplastic lesions. Among the neoplastic lesions, malignant lesions dominated the count. The highest number of cases was of astrocytoma followed by meningioma.

The age range considered was from the first year of life to 70 years. The most frequently involved age group was found to be that of 41-50 years, showing an incidence of 37 cases. The male‑to‑female ratio was 1.6:1. Studies conducted by Khonglah et al, Naik et al., and Hamdani et al. showed a ratio of 1.1:1, 2:1, and 1.2:0.8, respectively [2,5-7]. Despite some differences in ratios, all these studies show an inclination toward the male gender, which is similar to our study. The only oddity to the comprehensive distribution is meningioma, which was more common in females. This observation was supported by several studies, which could possibly be due to hormonal influence [8].

Most of the cases in our study presented with diversified clinical features, although headache was the most common presenting symptom in this series, which is supported by the findings of other studies [2,5-7]. Other clinical symptoms were seizures, altered sensorium, blurring of vision, and hydrocephalus.

The 2016 WHO Classification of CNS Tumors offers a makeshift histological grading system in which CNS tumors are classified into grades I to IV according to various factors, basically degree of malignancy. Several factors influence the systematic study for epidemiology of CNS tumors. In our study, astrocytoma was the most common tumor followed by meningioma, a finding in concordance with those of Khonglah et al., Agarwal et al., and Aryal [2,5,9]. However, various studies across India have reported meningioma to be the commonest tumor followed by astrocytoma [6,7]. Other frequently diagnosed tumors in our study were pituitary adenoma, schwannoma, and oligodendroglioma.

Among the 48 cases of astrocytoma, 22 cases were classified as low-grade astrocytoma and the remaining 26 cases as high-grade astrocytoma, which reflects the urgency to understand and evaluate the geomorphology of high-grade lesions that cause maximum fatality. Maximum cases, 18 cases out of 26 of high-grade astrocytoma, were diagnosed in the age group of 41-60 years, with a male dominance. Among the high-grade astrocytoma, anaplastic astrocytoma was found to be the most common entity (16 cases), followed by glioblastoma and gliosarcoma [10].

In the spectrum of CNS SOL biopsies that we received, extremely rare and unusual cases of extradural Rosai-Dorfman disease, PNET of D4-D5, SEGA, adenoid cystic carcinoma of orbit, ganglioglioma, anaplastic oligodendroglioma, gangliocytoma with pituitary adenoma, psammomatous meningioma of thoracic vertebrae, embryonal tumor NOS, meningioma with rhabdoid differentiation, gliosarcoma, anaplastic meningioma, metastatic squamous cell carcinoma, cavernous angioma, mucin-secreting metastatic carcinoma, and few other rare entities were also diagnosed.

**Figure 3:**
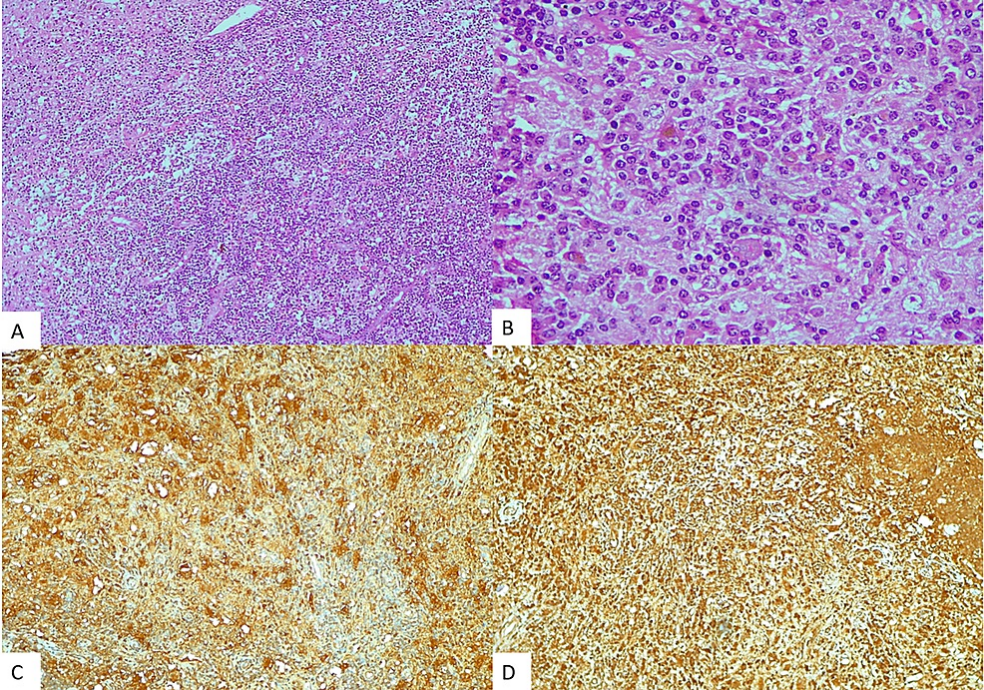
Photomicrograph of extradural Rosai-Dorfman disease. H&E x100 (A). H&E x400 (B). Diffuse CD68 positivity (C). Diffuse S100 positivity (D).

Rosai-Dorfman disease is a rare histiocytic disorder that confers classically with long-standing duration, self-limiting cervical lymphadenopathy [11]. With the help of histopathology, features such as large histiocytes were noted and were positive for IHC of CD68 and S100 protein and demonstrated characteristic emperipolesis. Thus, a diagnosis of Rosai-Dorfman disease could be made in our patient (Figure 3). The atypical clinical presentation and unusual involvement of multiple bones but not of lymph nodes is considered rare and aggressive [11,12].

Primary spinal PNETs are a rare entity with a poor prognosis [13]. Pathological findings were suggestive of PNET in our patient after correlation of histology and IHC showing CD99, FLi1 to be strongly positive. Ki-67 was 50% to 60%.

**Figure 4:**
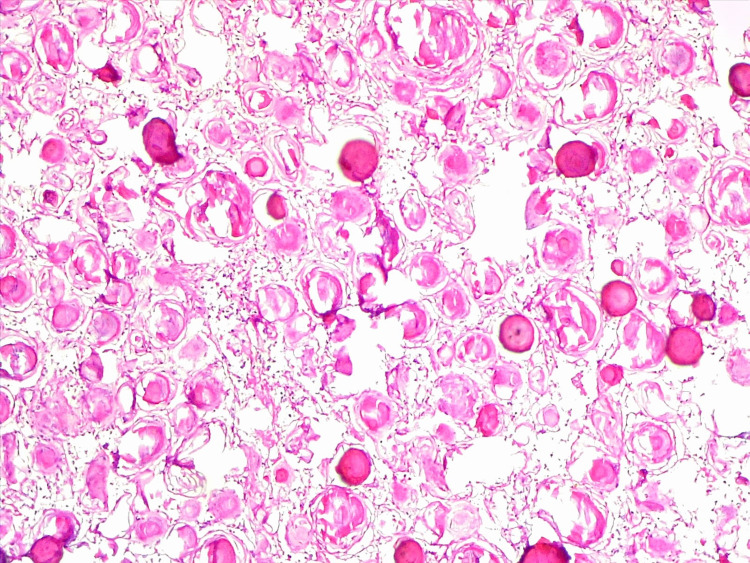
Photomicrograph of psammomatous meningioma showing numerous psammoma bodies, H&E x100.

Thoracic spinal psammomatous meningioma is also a rare subtype of meningioma. Psammomatous meningiomas are very rare tumors that complicate the surgical removal in certain cases, yet they are a curable pathology and carry a good prognosis if completely excised (Figure 4) [13,14].

**Figure 5:**
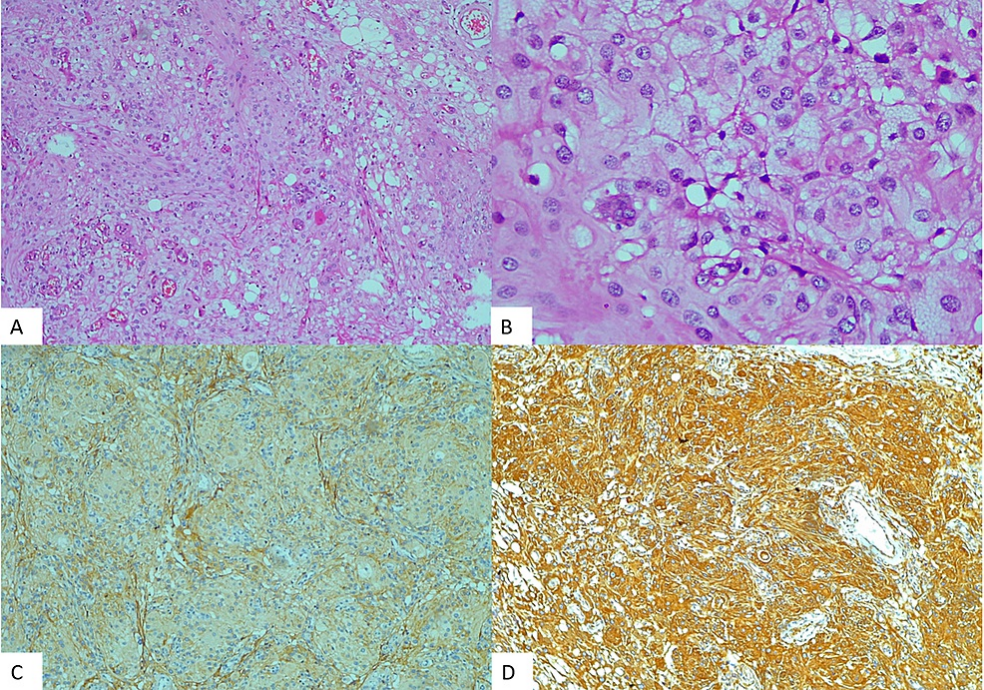
Photomicrograph of rhabdoid meningioma. H&E x100 (A). H&E x400 (B). Diffuse positivity EMA (C). Vimentin positivity (D). EMA, epithelial membrane antigen

Rhabdoid meningioma is a rare aggressive variant of meningioma and regarded as WHO grade III type. Histologically, it has abundant eosinophilic cytoplasm, cytoplasmic inclusion with eccentrically placed vesicular nuclei, and prominent nucleoli (Figure 5). It has a high recurrence rate and poor aftermath [15].

Gangliogliomas are rare tumors of the CNS. They can occur anywhere in the CNS but are usually found in the temporal lobe of children [16]. On histopathology examination, these were also confirmed by the presence of neoplastic ganglion cells and neoplastic glial component with IHCs showing both glial and neuronal components.

Anaplastic oligodendrogliomas usually develop secondarily via progression from a low-grade oligodendroglioma. The histological features are cellular, mitotically active, and diffusely infiltrating gliomas composed of neoplastic cells showing morphological features indicative of oligodendroglia cells, i.e., the classical fried egg appearance or perinuclear halo/clearing seen on histopathology as a fixation artifact [17,18].

Cerebrovascular malformations are non-neoplastic lesions resulting from focal anomalies in the development of cerebrospinal circulation. Many surveys suggest that cavernous angiomas are present in 0.5% population. They tend to be asymptomatic, but in 40% cases they can be symptomatic. Cavernous angiomas of hybrid variant are uncommon and remain exceptional. They can be either familial or sporadic [19,20]. In our patient, histopathology examination showed closely opposed vascular channels made up of hyaline fibrous wall with lining of single layer of endothelial cells and full of blood. There was no intervening cerebral parenchyma. Few areas of dystrophic calcification and ossification were seen. Normal cerebral parenchyma was noted at the periphery of the lesion. Some areas showed prominent congested capillaries. Histochemical stains such as Verhoffs and Masson trichrome stains showed the absence of elastic tissue and muscle in vessel walls, respectively.

**Figure 6:**
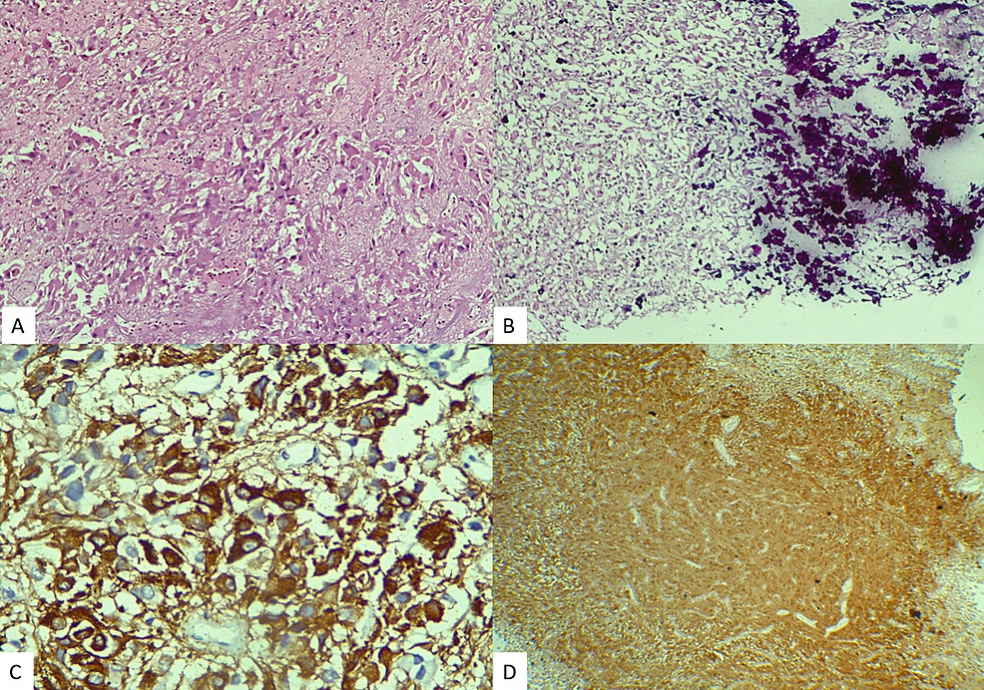
Photomicrograph showing sheets of large polygonal to elongate cells resembling astrocytes or ganglion cells with abundant, finely granular eosinophilic cytoplasm. H&E x100 (A). Extensive calcification, H&E x100 (B). Diffuse GFAP positivity (C). S100 positivity (D). GFAP, glial fibrillary associated protein

Histopathological examination of an intraventricular lesion revealed large polygonal to elongate cells resembling astrocytes or ganglion cells with abundant, finely granular eosinophilic cytoplasm, bright pink cellular processes, large round/oval nuclei, and prominent nucleoli [21]. Necrosis was also seen yet as per standard textbooks, which did not influence the upgrading of tumor. Final pathological diagnosis in our patient was SEGA with positive glial fibrillary associated protein (GFAP) and synaptophysin (Figure 6).

**Figure 7:**
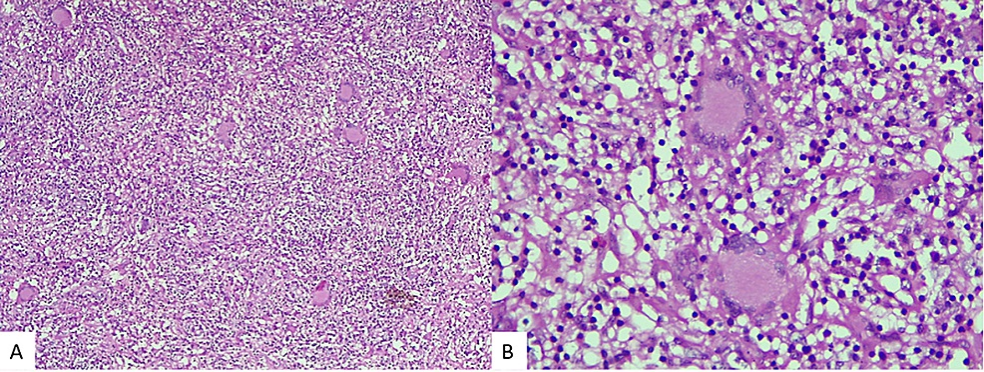
Photomicrograph showing formed granulomas comprising of non-caseating type of central necrosis at few places surrounded by epithelioid cells, lymphocytes, and many Langhans type of giant cells. H&E x100 (A). H&E x400 (B).

Sarcoidosis is an inflammatory granulomatous multisystem disease with an unknown etiology. Neurosarcoidosis is an extremely rare cryptogenic neuroinflammatory manifestation of sarcoidosis [22]. Histopathology examination of the lesion in our patient revealed many well-formed granulomas comprising of non-caseating type of central necrosis at few places surrounded by epithelioid cells, lymphocytes, and many Langhans type of giant cells (Figure 7). Special staining was negative for Ziehl-Neelsen, periodic acid Schiff, and Grocott methenamine silver, and a diagnosis of systemic sarcoidosis was made. Serum ACE levels were also elevated. The patient was referred to the Rheumatology Department for further management.

Gliosarcoma is an unusual subtype of glioblastoma characterized by biphasic cell population, constituting a definite, separate glial and sarcomatous differentiation on histology [23]. Histopathology of SOL in our patient showed tumor of biphasic differentiation with glial and sarcomatous components. Glial component showed high-grade nuclear atypia, mitosis, pseudo-palisading necrosis, and microvascular proliferation. Sarcomatous component showed densely packed spindle-shaped cells with nuclear atypia. Diagnosis was confirmed with positivity of IHC markers, which included GFAP, vimentin, and high Ki67 index.

**Figure 8:**
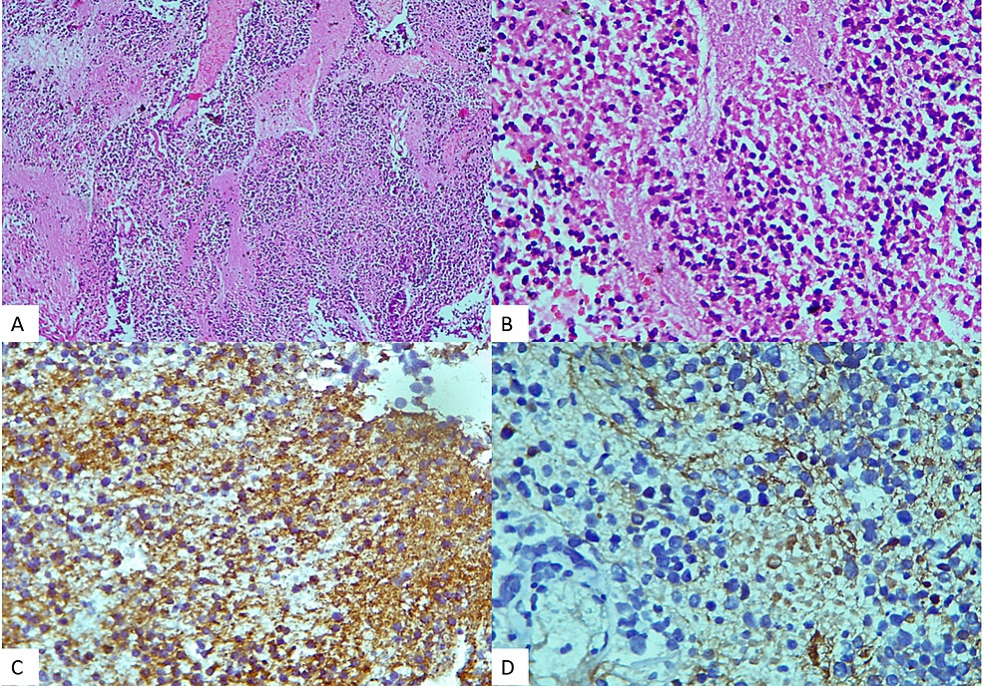
Photomicrograph showing the tumor comprised of diffusely lying cells with large round hyperchromatic nuclei and scant cytoplasm with extensive tumor necrosis. H&E x100 (A). H&E x400 (B). Synaptophysin positivity (C). Background positivity for GFAP (D). GFAP, glial fibrillary associated protein

CNS embryonal tumor NEC (not elsewhere classified) or NOS are assigned to those lesions in WHO Classification of CNS Tumors that lack a specific morphological and molecular alteration. They are poorly differentiated neoplasm with round-to-oval nuclei, high nuclear-to-cytoplasmic ratio, and frequent mitoses. Thus, an integrated, categorical layered diagnostic report is helpful for transparent and effective communication of relevant tumor characteristics. Histopathology of SOL in our patient showed normal neuroglial tissue with fragments of highly cellular tumor. The tumor comprised of diffusely lying cells with large round hyperchromatic nuclei and scant cytoplasm. Extensive tumor necrosis was also observed. There was no rosette formation observed. IHC revealed synaptophysin positivity in thin rim of cytoplasm and high Ki67 index of 70% (Figure 8). Thus, a diagnosis of CNS embryonal tumor NOS was offered and planned for molecular workup [24,25].

CNS neoplasms have been currently classified and graded on the basis of molecular cytogenetics in WHO CNS Neoplasms 2021, along with histology and IHCs [26]. Neurosurgical intervention is diverse, starting from simple observation, to minimal resection, wide local excision, radiation, chemotherapy, and combined management, all on the basis of tumor histological type, molecular profile, and tumor location. A high-grade tumor carries a poor prognosis, whereas a low-grade tumor has a better prognosis and outcome [27,28]. The major limitation of our study was that we were unable to classify the lesions as per molecular cytogenetics due to lack of resources and expertise. However, in near future, we aim forward to conduct a study with a larger group of patients and analyze them on the basis of histology, IHC, and cytogenetics, which will have a greater impact on the research.

## Conclusions

Conclusions are evident with evidence provided and summarizes that CNS lesions are heterogeneous, comprising a large spectrum of different tumors, inflammatory lesions, and infections and that these entities have further distinct biological background and disease course. Histopathological study helps in knowing their epidemiology and burden of disease in the community. Likewise, we observed a male predominance, with those in the age group of 41-50 years with maximum incidence, and highest tumor burden in astrocytoma followed by meningiomas. From a practical point of view, an accurate diagnosis of brain tumor is possible after careful assessment of histomorphological features along with clinical and radiological imaging findings. Though established histopathological examination is the mainstay or gold standard for diagnosis, IHC also has also played a major role in subcategorizing the exact diagnosis and improvising the field of neuropathology. The present study thus reflects the diversity of histopathological spectrum of CNS lesions from our center. In-depth studies from across various hospitals are required to have representative data on the incidence of CNS lesions in our country. These could then be used to provide the baseline data to better understand the epidemiological profile and etiology of primary brain tumors so that prompt neurosurgical intervention could benefit the patients in the long run.
